# Risk prediction model for thrombosis in leukemia patients: a systematic review

**DOI:** 10.3389/fonc.2026.1775197

**Published:** 2026-02-26

**Authors:** Minlan Ye, Yingjie Tian, Liang Su, Fang Ye, Jie Wu

**Affiliations:** 1 Guang’anmen Hospital, China Academy of Chinese Medical Sciences, Beijing, China; 2 Chuiyangliu Hospital Affiliated with Tsinghua University, Beijing, China

**Keywords:** leukemia, predictor, risk prediction model, systematic review, thrombosis

## Abstract

Thrombosis represents a significant complication in leukemia patients, associated with treatment interruption and reduced survival outcomes. Although multiple risk prediction models have been developed, their methodological quality and applicability remain uncertain. This review aims to evaluate existing risk prediction models for thrombosis in patients with leukemia. We conducted comprehensive literature searches across nine databases from the inception to August 4, 2025. Two reviewers independently performed study selection, data extraction, and quality assessment using the CHARMS checklist and PROBAST tool. Of 1825 initially identified records, 14 studies comprising 16 prediction models were included. Development cohorts ranged from 102 to 1252 participants. Model discrimination measured by AUC/C-index varied between 0.641-0.917. Internal validation was performed in nine studies, while only one conducted external validation. Key predictors included central venous catheter placement, prior history of thrombosis, D-dimer levels, platelet count, white blood cell count, the Eastern Cooperative Oncology Group (ECOG) score, chemotherapy/radiotherapy, comorbidities, type of leukemia, use of hemostatic drugs, and age. All studies were rated high risk of bias, and five raised major concerns regarding applicability. Sensitivity analyses excluding chronic leukemia studies, excluding non-English publications and excluding dissertations yielded consistent overarching conclusions. In summary, current models often report moderate to good apparent discrimination, but are limited by methodological shortcomings and inadequate validation. All models should be considered exploratory and not ready for routine clinical use without prospective external validation. Future research should prioritize prospective, multicenter cohorts with standardized outcome adjudication and rigorous internal/external validation across diverse leukemia subtypes.

## 1 Introduction

Leukemia is a group of hematologic malignancies, primarily classified into acute leukemia (AL) and chronic leukemia. Acute leukemia includes acute lymphoblastic leukemia (ALL) and acute myeloid leukemia (AML). Among these, acute promyelocytic leukemia (APL) represents a distinct and rare subtype of AML. Thrombosis is one of the critical complications requiring vigilance in leukemia patients, leading to multiple negative impacts. These include chemotherapy interruption and increased bleeding risk due to anticoagulant therapy, post-thrombotic syndrome and various sequelae, as well as shortened overall survival and progression-free survival (1, 2). From the perspective of thrombosis risk in the overall cancer population, thrombosis ranks as the second leading cause of mortality and morbidity, surpassed only by the cancer itself (3–5). Data indicate that cancer patients face a four to ninefold increased risk of thrombosis compared to the general population (6–8), and cancer is associated with 20-30% of initial venous thrombotic events (9). Moreover, the risk of thrombosis in hematologic malignancies is similar to or even higher than that in solid tumor patients (10, 11). Specifically, regarding leukemia subtypes, the thrombosis incidence rate in ALL is 1.7-16% (12–22), in AML (excluding APL) it is 1.6-14.6% (23–27), while APL, due to the potential coexistence of thrombotic and hemorrhagic manifestations (28), has a thrombosis incidence rate of 5.2-20.6% (29–32). Thrombotic events in leukemia are predominantly venous thromboembolism (VTE), including deep vein thrombosis, pulmonary embolism, and central venous catheter-related thrombosis, while arterial thromboembolism (ATE) has been less studied (33, 34). Therefore, early identification of leukemia patients at high thrombosis risk and implementing targeted prevention are key to reducing the thrombotic burden and improving clinical outcomes.

Currently, the commonly used risk prediction models for cancer-associated thrombosis are the Khorana score (35) and the Vienna Cancer and Thrombosis Study (CATS) score (36). The Khorana score includes five predictive factors: cancer site, platelet count ≥350×10^9^/L, hemoglobin <100 g/L, and/or use of erythropoiesis-stimulating agents, white blood cell count >11×10^9^/L, and body mass index ≥35 kg/m^2^. The CATS score adds two biomarkers, soluble P-selectin and D-dimer, to the factors in the Khorana score. However, the development populations for both models did not include leukemia patients. Furthermore, the universal features of leukemia, such as anemia and leukocytosis, may limit the risk assessment performance of these models (37). Consequently, developing thrombosis risk prediction models specific to leukemia is crucial.

In recent years, although risk prediction models for thrombosis in leukemia patients have been successively proposed, providing new tools for clinical risk stratification, a comprehensive assessment of their quality and applicability has been lacking. This study aims to systematically review the development methods, predictive indicators, and validation results of thrombosis risk prediction models in leukemia patients, grade the evidence, and propose directions for optimization. The findings will inform clinical decision-making and guide the design of subsequent research initiatives.

## 2 Methods

The study protocol was registered on the International Prospective Register of Systematic Reviews (PROSPERO; Registration number: CRD420251146253).

### 2.1 Search strategy

A systematic literature search was performed across nine databases: PubMed, Web of Science, Embase, The Cochrane Library, Cumulative Index to Nursing and Allied Health Literature (CINAHL), China National Knowledge Infrastructure (CNKI), Wanfang Database, China Science and Technology Journal Database (VIP), and Chinese Biomedical Literature Database (CBM), to identify relevant studies published from the inception of each database until August 4, 2025. The search utilized the following keywords: “leukemia”, “thrombosis”, “embolism”, “thromboembolism”, “risk prediction model”, “risk factor”, “predictor”, “model”, “risk score”, “risk assessment”. Two researchers independently conducted the literature search, and any disagreements were resolved by a third researcher. The comprehensive search strategies for the various databases are outlined in the ‘**Supplementary Material**’. To illustrate, a systematic search of the PubMed database was conducted, following these steps:

#1 (“Leukemia”[Mesh]) OR (((((Leukaemia[Title/Abstract]) OR (Leukemia[Title/Abstract])) OR (Leukemias[Title/Abstract])) OR (Leucocythaemia[Title/Abstract])) OR (Leucocythemia[Title/Abstract])).

#2 (((((“Embolism and Thrombosis”[Mesh]) OR (Embolism[Title/Abstract] AND Thrombosis[Title/Abstract])) OR (Thrombosis[Title/Abstract] AND Embolism[Title/Abstract])) OR (Embolism[Title/Abstract])) OR (Thromboembolism[Title/Abstract])) OR (Thrombosis[Title/Abstract]).

#3 (((((Risk prediction model[Title/Abstract]) OR (Risk factor[Title/Abstract])) OR (Predictor[Title/Abstract])) OR (Model[Title/Abstract])) OR (Risk Score[Title/Abstract])) OR (risk assessment[Title/Abstract]).

#4 #1 AND #2 AND #3.

Furthermore, following the recommendations of the Checklist for Critical Appraisal and Data Extraction for Systematic Reviews of Prediction Modelling Studies (CHARMS) (38), this review employed the PICOTS framework to systematically delineate the population, index, comparator, outcome, timing, and setting in this review. The key elements of this systematic review are as follows:

P (Population): Patients with leukemia, including both acute and chronic leukemia.

I (Index): Development of a risk prediction model for thrombosis in leukemia patients.

C (Comparator): Not applicable.

O (Outcome): Occurrence of thrombosis, including venous thromboembolism and/or arterial thrombosis.

T (Timing): No restrictions; includes both short-term and long-term prediction horizons.

S (Setting): Inpatient or outpatient settings.

### 2.2 Inclusion and exclusion criteria

Studies were included if they met the following criteria: (1) Study population consisted of patients diagnosed with any subtype of leukemia (acute and chronic leukemia); (2) The study focused on developing or improving a risk prediction model for thrombosis in leukemia patients; (3) The primary outcome was thrombotic events (including venous thromboembolism and/or arterial thrombosis); (4) Study designs comprised cohort, case-control, cross-sectional, and registry-based studies.

Exclusion criteria were as follows: (1) The final prediction model contained fewer than two predictors; (2) Case reports, systematic reviews, conference abstracts, and basic science research; (3) Duplicate publications and studies with incomplete data.

### 2.3 Study selection and screening

Duplicate records were first eliminated using EndNote 20, followed by manual inspection to identify any remaining duplicates. Articles were then screened by title and abstract. The full texts of potentially relevant studies were reviewed to confirm eligibility. Reference lists of all included studies were examined to ensure a thorough retrieval of relevant literature. Two investigators independently carried out the screening process and cross-verified results. Disagreements were resolved through consensus or arbitration by a third reviewer.

### 2.4 Data collection

A standardized data extraction form, based on the CHARMS checklist, was used to collect data in two main categories: (1) Basic study characteristics: first author, year of publication, country, study design, study population, data source, and outcome definition. (2) Model-specific information: variable selection method, strategy for handling missing data, method for handling continuous variables, model development strategy, type of model validation, model performance metrics, final predictors retained, and model presentation format. Data were independently extracted by two researchers and then cross-checked. Any discrepancies were settled through discussion or by consulting a third researcher.

### 2.5 Quality assessment

The Prediction Model Risk of Bias Assessment Tool (PROBAST) (39) was used to assess the risk of bias and the applicability of the included prediction modeling studies. This tool assesses the risk of bias across four domains: participants, predictors, outcome, and analysis. It also assesses the applicability of the first three domains. The tool comprises 20 signaling questions, each answered as “yes,” “probably yes,” “no,” “probably no,” or “no information.” Reviewers answered these questions based on their judgment. The overall risk of bias was rated as ‘low’ only if all domains were rated as ‘low risk.’ It was rated as ‘high’ if at least one domain was rated as ‘high risk.’ If at least one domain was rated as ‘unclear,’ and no domains were rated as ‘high risk’ or ‘low concern’ regarding applicability, the overall rating was ‘unclear.’ Two reviewers independently performed the assessments, with disagreements resolved through discussion or a third researcher.

### 2.6 Data synthesis

A descriptive analysis of the included studies was conducted. Key findings were summarized in tables covering: (1) basic study characteristics: including study design, participants, data sources, outcome definition, and outcome measures; (2) Model development and validation details: modeling technique, candidate variables, event numbers, missing data management, and final predictors; (3) Model performance and interpretability: discrimination (e.g., AUC, C-statistic), calibration (via plots and Hosmer-Lemeshow test where available), validation approaches, and presentation format. To assess the robustness of our narrative synthesis, we conducted sensitivity analysis as follows: (1) excluding studies enrolling chronic leukemia patients; (2) excluding articles not published in English. (3) excluding non-peer-reviewed dissertations.

## 3 Results

### 3.1 Study selection

**Figure 1:**
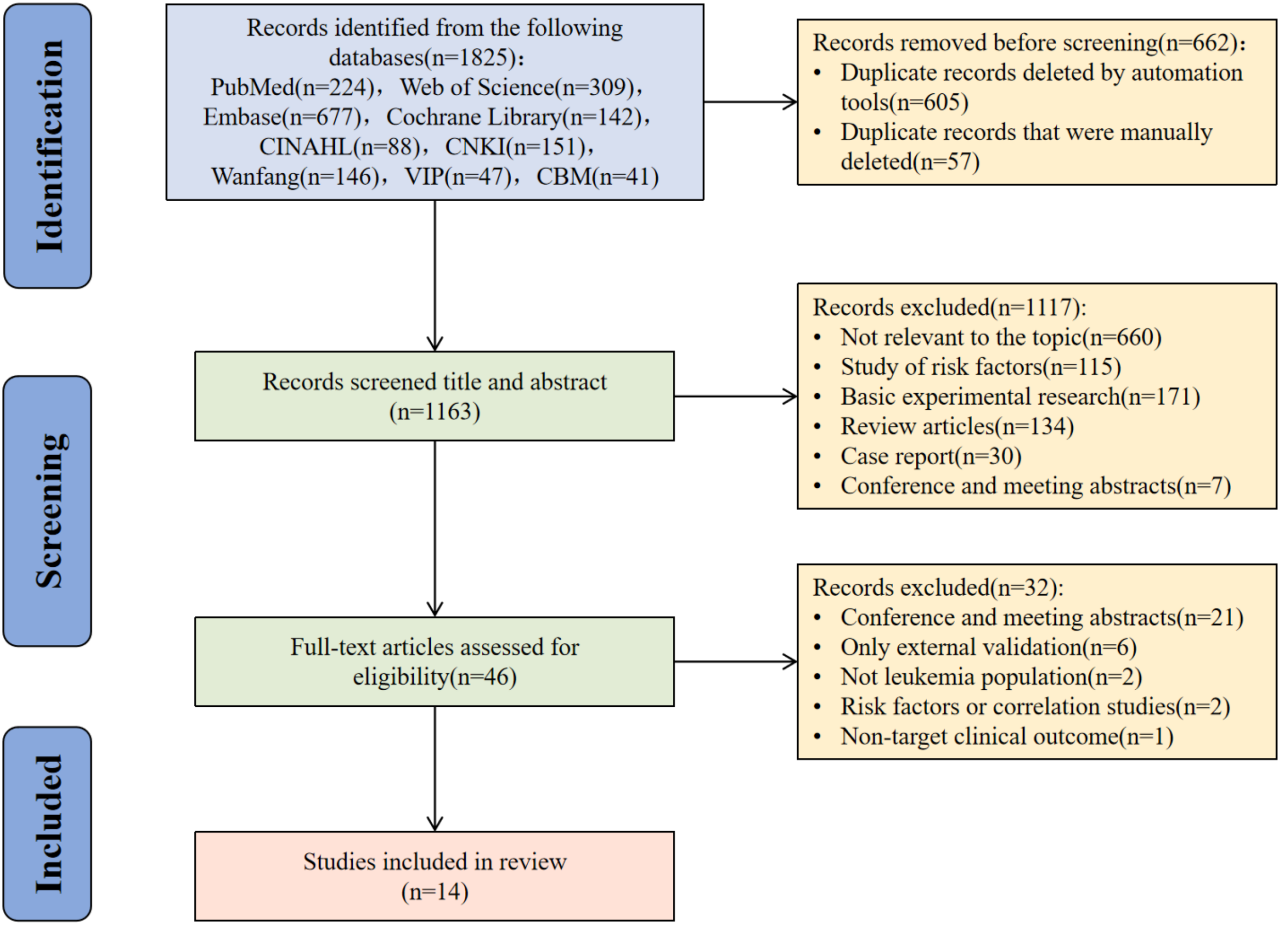
Study selection process based on the PRISMA guidelines.

**Figure 1** illustrates the literature selection process, which followed to the Preferred Reporting Items for Systematic Reviews and Meta-Analyses (PRISMA) 2020 guidelines. The initial search retrieved 1825 records from the targeted databases. Following the removal of 662 duplicates, 1163 records underwent title and abstract screening. This step resulted in the exclusion of 1117 records. The remaining 46 articles underwent full-text review for eligibility, of which 32 were excluded, leaving 14 studies for final inclusion in this systematic review.

### 3.2 Basic characteristics of included models

The 14 studies included in this review consisted of 9 English (27, 40–42, 45, 47, 49, 51, 52) and 5 Chinese (43, 44, 46, 48, 50) publications. These studies were published between 2010 and 2025, except for 1 study (40), and the remaining 13 were published in the past 5 years (27, 41–52). Among the included studies, 7 were conducted in China (43, 44, 46, 48, 50–52), 1 in Germany and France (40), 1 in the Nordic and Baltic countries (41), 1 in Canada (42), 1 in Italy (27), 1 in Israel (45), 1 in Thailand (47), and 1 in Serbia (49). Regarding study design, 9 were retrospective cohort studies (27, 42, 43, 45, 47, 49–52), 3 were case-control studies (44, 46, 48), and 2 were prospective cohort studies (40, 41). All studies focused on leukemia patients, including ALL, AML, APL, and CML. The primary outcomes included VTE-only outcomes (40, 42, 44, 46, 49), composite thrombotic outcomes defined as VTE and/or ATE (27, 41, 47, 52), catheter-related thrombosis (CRT), including peripherally inserted central catheter (PICC)-related thrombosis (43, 45, 50, 51), and cerebral infarction (48). VTE was primarily diagnosed using imaging techniques such as ultrasound, MRI, CT, venography, and CTPA. Myocardial infarction diagnosis relied on clinical presentation, electrocardiography, and cardiac biomarkers, while other ATEs were diagnosed using CT or MRI. The basic characteristics of the included studies are shown in **Table 1**.

**Table 1:** Basic characteristics of inclusion in the literature.

Authors(year)	Country	Study design	Participants	Data source	Outcome	Outcome measure
Mitchell et al. (2010) (40)	Germany, France	Prospective cohort study	ALL	Children’s hematology centers in several European countries such as Germany and France	VTE	Standard imaging methods
Jarvis et al. (2019) (41)	The Nordic and Baltic countries	Prospective cohort study	ALL	Patients with ALL included in the NOPHO ALL2008 protocol	VTE or ATE	Imaging methods, such as ultrasound, MRI, and venography
Al-Ani et al. (2020) (42)	Canada	Retrospective cohort study	Acute leukemia	London Health Sciences Centre, a tertiary care center in London, Ontario, Canada	VTE	A compression ultrasound, contrast venography, ventilation-perfusion lung scan, CTPA
He et al. (2022) (43)	China	Retrospective cohort study	Acute leukemia	Pediatrics Department of the First People’s Hospital of Guangyuan City, Sichuan Province	PICC-related thrombosis	Transcatheter site ultrasound
Pang et al. (2022) (44)	China	Case-control study	ALL	Gansu Provincial Cancer Hospital	Deep venous thromboembolism	MRI, ultrasound etc.
Paterno et al. (2022) (27)	Italy	Retrospective cohort study	AML(non-APL)	Hematology, Department of Biomedicine and Prevention, University Tor Vergata	VTE or ATE	VTE: Doppler ultrasonography, CT, or MRI; Myocardial infarction(MI): clinical, enzymatic and electrocardiographic criteria; ATE other than MI: CT
Perek et al. (2022) (45)	Israel	Retrospective cohort study	AML; AML(non-APL)	The Rambam Health Care Campus	Catheter-related thrombosis (CRT)	Doppler ultrasound
Yang et al. (2023) (46)	China	Case-control study	Acute leukemia(non-APL)	Zhengzhou University Affiliated Cancer Hospital, Henan University of Science and Technology First Affiliated Hospital	VTE	Imaging examination
Owattanapanich et al. (2023) (47)	Thailand	Retrospective cohort study	Acute leukemia	Faculty of Medicine Siriraj Hospital, Mahidol University	VTE or ATE	Compression Doppler ultrasonography, CTPA, or CT
Li et al. (2024) (48)	China	Case-control study	AML(non-APL)	Zhengzhou University Affiliated Tumor Hospital, Zhengzhou University Affiliated People’s Hospital	Cerebral infarction(CI)	Head CT or MRI examination
Mitrovic et al. (2024) (49)	Serbia	Retrospective cohort study	AML (non-APL)	The Clinic for Hematology at the University Clinical Center of Serbia	VTE	Compression ultrasound, CTPA
Zhang et al. (2025) (50)	China	Retrospective cohort study	Acute leukemia	The First Affiliated Hospital of Zhengzhou University	PICC-related thrombosis	Doppler ultrasound or CT examination
Fu et al. (2025) (51)	China	Retrospective cohort study	ALL, AML, CML	Tongji Hospital, Tongji Medical College, Huazhong University of Science and Technology	PICC-related thrombosis	Doppler ultrasonography, MRI, or venography
Hao et al. (2025) (52)	China	Retrospective cohort study	APL	The Second Hospital of Shanxi Medical University	VTE or/and ATE	VTE: vascular Doppler ultrasound or CTPA; ATE: clinical symptoms, physical examination findings, ECG, cardiac biomarkers, CT or MRA

ALL, acute lymphoblastic leukemia; AML, acute myeloid leukemia; APL, acute promyelocytic leukemia; CML, chronic myeloid leukemia; TE, thromboembolism; VTE, venous thromboembolism; ATE, arterial thromboembolism; PICC, peripherally intravenous central catheter; MRI, magnetic resonance imaging; CT, computed tomography; CTPA, computed tomography pulmonary angiography; MRA, magnetic resonance angiography.

### 3.3 Model development and predictors

**Figure 2:**
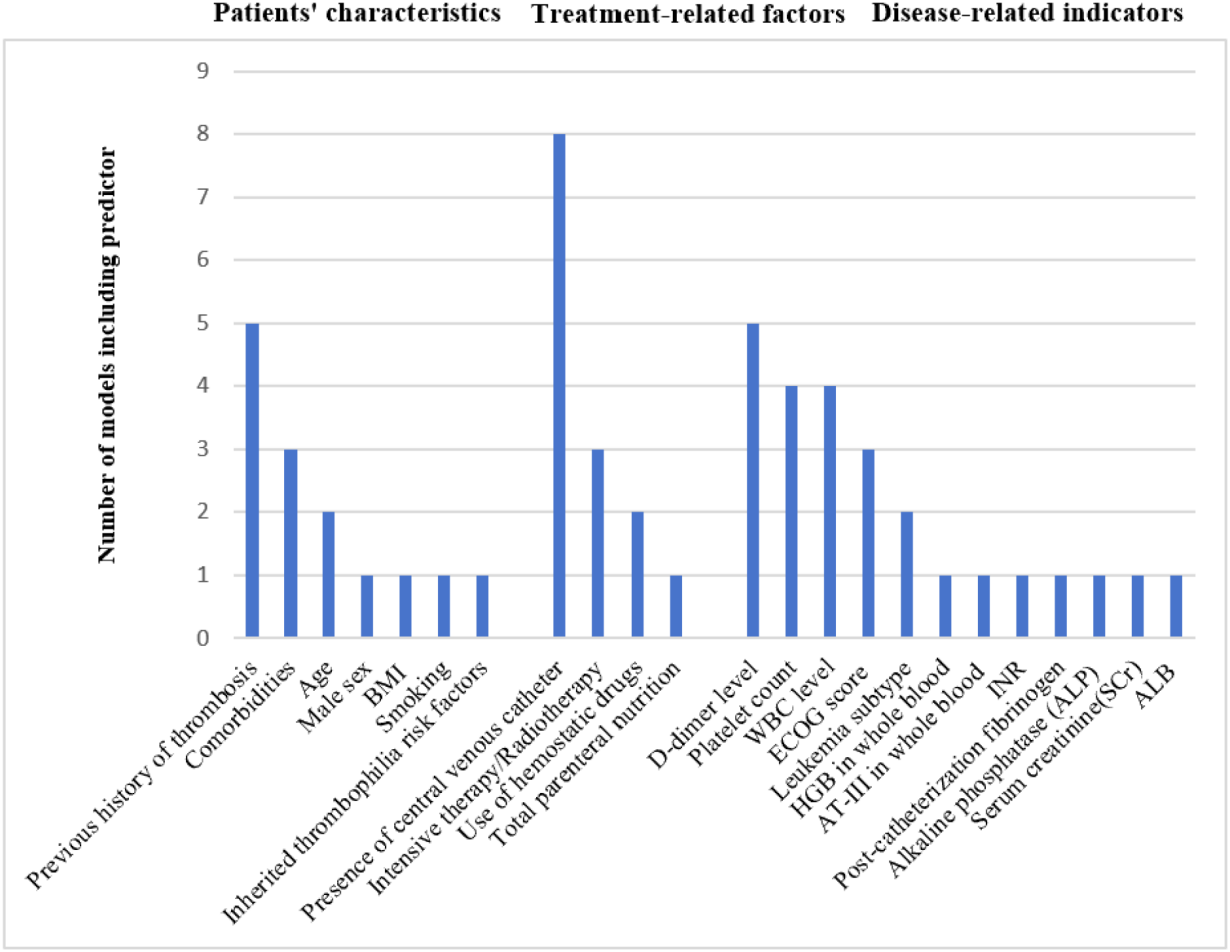
Main categories of predictors included in developed models. ECOG score, Eastern Cooperative Oncology Group score; INR, International normalized ratio; AT-III, Antithrombin; ALB, albumin.

In the included studies, the development set sample sizes ranged from 102 to 1252 participants, with the number of outcome events ranging from 16 to 116. For studies reporting validation (40, 51), sample sizes were 339 and 155, with corresponding event numbers of 19 and 30. The number of candidate predictors considered ranged from 3 to 54. In 11 studies, continuous variables were converted into categorical variables (27, 41–47, 50–52). Regarding missing data, 9 studies directly excluded cases with missing values directly (40, 41, 43, 44, 47, 49–52), while 5 studies did not specify the extent or handling of missing data (27, 42, 45, 46, 48). The final models incorporated between 2 and 6 predictors. The most commonly identified predictors included central venous catheter placement, prior history of thrombosis, D-dimer levels, platelet count, white blood cell count, Eastern Cooperative Oncology Group (ECOG) score, chemotherapy/radiotherapy, comorbidities, leukemia type, use of hemostatic drugs, and age, as detailed in **Figure 2**. A total of 16 models were developed across the 14 studies. Techniques used for model development included logistic regression (27, 42–52), Cox regression (40, 41), and random forest (50), with logistic regression being the most common method. Key characteristics and predictors of the developed models are detailed in **Table 2**.

**Table 2:** Overview of the information of the included prediction models.

Author(s) (year)	Sample size (development cohorts/validation cohorts)		EPV	Candidate variables		Missing data		Final predictors	Modeling methods (number of models)
	Number	Outcome cases		Number	Continuous variable processing method	Number	Method		
Mitchell et al. (2010) (40)	456/339	34/19	11.33	3	Left unaltered	Not report	Excluded	Treatment, presence of central venous catheter, thrombophilic abnormalities	CR(1)
Jarvis et al. (2019) (41)	1252/-	89/-	22.25	4	Discretized	7	Excluded	F11 rs2036914, FGG rs2066865	CR(1)
Al-Ani et al. (2020) (42)	501/-	77/-	3.85	20	Discretized	126	Not report	Previous history of venous thromboembolism, lymphoblastic leukemia, platelet count > 50×10^9^/L at the time of diagnosis	LR(1)
He et al. (2022) (43)	184/-	38/-	2.11	18	Discretized	Not report	Excluded	Catheter placement, catheter-related infection, use of hemostatic drugs, and the D-D level 15 days after catheter placement	LR(1)
Pang et al. (2022) (44)	102/-	51/-	3.4	15	Discretized	Not report	Excluded	Radiotherapy, HGB in whole blood < 100g/L, WBC in whole blood ≥ 10×10^9^/L, D-dimer in whole blood ≥ 0.55mg/L, AT-III in whole blood < 75%	LR(1)
Paterno et al. (2022) (27)	300/-	36/-	1.64	22	Discretized	Not report	Not report	Comorbidities, platelets count >50 × 10^9^/L, a previous history of VTE	LR(1)
Perek et al. (2022) (45)	Entire AML:632/- AML(non-APL):587/-	Entire AML:64/- AML(non-APL):55/-	Entire AML :2.13 AML(non-APL):1.96	Entire AML :30 AML(non-APL):28	Discretized	Not report	Not report	Entire AML :APL, prior VTE, BMI and platelet counts <100 × 10^9^/L AML(non-APL):BMI, prior VTE, COPD and an initial platelet count <100 × 10^9^/L	LR(2)
Yang et al. (2023) (46)	290/-	116/-	6.11	19	Discretized	Not report	Not report	Previous history of VTE/coronary heart disease/stroke, ECOG score of≥2, WBC> 11×10^9^/L, and ALB < 35g/L	LR(1)
Owattana-panich et al. (2023) (47)	261/-	16/-	2.67	6	Discretized	Not report	Excluded	D-dimer>7000 µg FEU/L, platelet>40× 10^9^/L, and white blood cell level>15× 10^9^/L	LR(1)
Li et al. (2024) (48)	207/-	82/-	3.15	26	Left unaltered	Not report	Not report	Age, WBC level, ECOG score≥2, prognostic high-risk group, and co-infection	LR(1)
Mitrovic et al. (2024) (49)	626/-	72/-	1.89	38	Left unaltered	List the missing value(%) for each item	Excluded	Male sex, prior history of thrombotic events, INR, ECOG score, CVL, and intensive therapy	LR(1)
Zhang et al. (2025) (50)	120/-	35/-	2.92	12	Discretized	Not report	Excluded	Puncture site, puncture times, catheter indwelling time, catheter- related complications, use of anticoagulant drugs and D-dimer level	LR, RF(2)
Fu et al. (2025) (51)	364/155	68/30	1.26	54	Discretized	Not report	Excluded	Leukemia, number of catheters, history of catheterization, total parenteral nutrition, post-catheterization D-dimer, and post-catheterization fibrinogen	LR(1)
Hao et al. (2025) (52)	306/-	16/-	0.5	32	Discretized	39	Excluded	Age, smoking, alkaline phosphatase >125U/L, and serum creatinine>62µmol/L	LR(1)

LR, logistic regression; CR, cox regression; RF, random forest; ECOG score:Eastern Cooperative Oncology Group score.

### 3.4 Model performance and presentation

The area under the curve (AUC) and the C-index are commonly reported as indicators of model discrimination. In the development set, they range from 0.641 to 0.917, while in the validation set, only one study reported a value of 0.794 (51). Two studies did not report the AUC or C-index (40, 41), but one of them evaluated the performance using specificity, sensitivity, and accuracy (40). Nine studies reported calibration via the Hosmer-Lemeshow test and/or calibration curves (27, 42, 44–46, 48, 49, 51, 52). Internal validation was the most frequently used validation method across all included studies. Specifically, nine studies performed internal validation using bootstrapping (27, 42, 45–49, 51, 52), while only one study conducted external validation (40). Various methods were used to present the prediction models, including risk scores, formulas, and nomograms. The performance and presentation of the prediction models are detailed in **Table 3**.

**Table 3:** Performance and presentation of prediction models for thrombosis in leukemia.

Author(s) (year)	Discrimination—AUC/C-index (95% CI) in the development cohorts	Discrimination—AUC/C-index (95% CI) in the validation cohorts	Calibration	Model validation—Internal validation	Model validation—External validation	Model presentation
Mitchell et al. (2010) (40)	Not report	Not report	Not report	None	Geographical validation	Risk score
Jarvis et al. (2019) (41)	Not report	Not report	Not report	None	None	Risk score
Al-Ani et al. (2020) (42)	0.664(0.590-0.738)	Not report	Hosmer-Lemeshow test	Bootstrap	None	Risk score
He et al. (2022) (43)	0.917(0.866-0.954)	Not report	Not report	None	None	Formula
Pang et al. (2022) (44)	0.876(0.796-0.933)	Not report	Hosmer-Lemeshow test	None	None	Formula
Paterno et al. (2022) (27)	0.641(0.534-0.747)	Not report	Hosmer-Lemeshow test	Bootstrap	None	Risk score
Perek et al. (2022) (45)	Entire AML: 0.698(0.626-0.771) AML(non-APL):0.711 (0.635-0.789)	Not report	Hosmer-Lemeshow test	Bootstrap	None	Formula
Yang et al. (2023) (46)	0.754(0.698-0.811)	Not report	Hosmer-Lemeshow test and calibration curve	Bootstrap	None	Nomogram and formula
Owattanapanich et al. (2023) (47)	0.83(0.75-0.90)	Not report	Not report	Bootstrap	None	Risk score
Li et al. (2024) (48)	0.809(0.750-0.867)	Not report	Hosmer-Lemeshow and calibration curve	Bootstrap	None	Nomogram and formula
Mitrovic et al. (2024) (49)	0.68(0.61-0.74)	Not report	Calibration curve	Bootstrap	None	Nomogram
Zhang et al. (2025) (50)	RF:0.860(0.785-0.916) LR:0.775(0.690-0.846)	Not report	Not report	None	None	Formula and random forest model
Fu et al. (2025) (51)	0.844(0.787-0.900)	0.794(0.698-0.890)	Hosmer-Lemeshow test and calibration curve	Bootstrap	None	Nomogram
Hao et al. (2025) (52)	0.875(0.782-0.968)	Not report	Hosmer-Lemeshow test and calibration curve	Bootstrap	None	Nomogram

### 3.5 Sensitivity analyses

We performed three sensitivity analyses to assess the robustness of our descriptive synthesis. First, chronic leukemia was represented only by CML within one mixed-subtype cohort (51) (**Supplementary Table S1**). The remaining evidence base showed a similar range of reported discrimination and the same overarching limitations in validation. Second, excluding articles not published in English (43, 44, 46, 48, 50) (**Supplementary Table S2**) reduced the number of included models, but the overall interpretation remained unchanged. Most models reported moderate-to-good discrimination in development cohorts, while calibration reporting and external validation were limited. Third, excluding non–peer-reviewed dissertations (46, 48) (**Supplementary Table S3**) did not materially alter the key conclusions. Across all restricted analyses, the central findings were consistent that high risk of bias and insufficient validation were common. The current models should be considered exploratory and not ready for routine clinical implementation.

### 3.6 Subtype-stratified summary of model performance

Given clinically meaning differences in thrombosis mechanisms and management across leukemia subtypes, we summarized model characteristics and performance stratified by subtype (**Table 4**). Briefly, subtype-specific models were available for ALL, AML (including non-APL cohorts), and APL, while evidence explicitly targeting CML was scarce and primarily embedded within mixed-subtype cohorts. Across subtypes, discrimination was variably reported and calibration was inconsistently assessed; internal validation was more common than external validation. **Table 4** provides an at-a-glance overview of outcomes modeled, predictors retained, validation strategies, and reported discrimination by subtype, highlighting important evidence gaps for transportability and clinical implementation.

**Table 4:** Model performance and characteristics stratified by leukemia subtype.

Leukemia subtype	Author(s) (year)	Country	Outcome	Modeling methods (no. of models)	Final predictors	Discrimination (development)	Discrimination (validation)	Calibration	Validation	Model presentation
ALL	Mitchell et al. (2010) (40)	Germany, France	VTE	CR (1)	Treatment, presence of central venous catheter, thrombophilic abnormalities	NR	NR	NR	Geographical validation	Risk score
ALL	Jarvis et al. (2019) (41)	The Nordic and Baltic countries	VTE or ATE	CR (1)	F11 rs2036914, FGG rs2066865	NR	NR	NR	None	Risk score
ALL	Pang et al. (2022) (44)	China	Deep venous thromboembolism	LR(1)	Radiotherapy, HGB in whole blood < 100g/L, WBC in whole blood ≥ 10×10^9^/L, D-dimer in whole blood ≥ 0.55mg/L, AT-III in whole blood < 75%	0.876(0.796-0.933)	NR	Hosmer-Lemeshow test	None	Formula
AML (non-APL)	Paterno et al. (2022) (27)	Italy	VTE or ATE	LR (1)	Comorbidities, platelets count >50 × 10^9^/L, a previous history of VTE	0.641(0.534-0.747)	NR	Hosmer-Lemeshow test	Bootstrap	Risk score
AML (overall)	Perek et al. (2022) (45)	Israel	Catheter-related thrombosis (CRT)	LR (Model 1 of 2)	APL, prior VTE, BMI and platelet counts <100 × 10^9^/L	0.698(0.626-0.771)	NR	Hosmer-Lemeshow test	Bootstrap	Formula
AML (non-APL)	Perek et al. (2022) (45)	Israel	Catheter-related thrombosis (CRT)	LR (Model 2 of 2)	BMI, prior VTE, COPD and an initial platelet count <100 × 10^9^/L	0.711(0.635-0.789)	NR	Hosmer-Lemeshow test	Bootstrap	Formula
AML(non-APL)	Li et al.(2024) (48)	China	Cerebral infarction(CI)	LR(1)	Age, WBC level, ECOG score≥2, prognostic high-risk group, and co-infection	0.809(0.750-0.867)	NR	Hosmer-Lemeshow and calibration curve	Bootstrap	Nomogram and formula
AML (non-APL)	Mitrovic et al. (2024) (49)	Serbia	VTE	LR(1)	Male sex, prior history of thrombotic events, INR, ECOG score, CVL, and intensive therapy	0.68(0.61-0.74)	NR	Calibration curve	Bootstrap	Nomogram
APL	Hao et al. (2025) (52)	China	VTE or/and ATE	LR(1)	Age, smoking, alkaline phosphatase >125U/L, and serum creatinine>62µmol/L	0.875 (0.782–0.968)	NR	Hosmer-Lemeshow test and calibration curve	Bootstrap	Nomogram
Acute leukemia	Al-Ani et al. (2020) (42)	Canada	VTE	LR(1)	Previous history of venous thromboembolism, lymphoblastic leukemia, platelet count > 50×10^9^/L at the time of diagnosis	0.664(0.590-0.738)	NR	Hosmer-Lemeshow test	Bootstrap	Risk score
Acute leukemia	He et al.(2022) (43)	China	PICC-related thrombosis	LR(1)	Catheter placement, catheter-related infection, use of hemostatic drugs, and the D-D level 15 days after catheter placement	0.917(0.866-0.954)	NR	Not report	None	Formula
Acute leukemia (non-APL)	Yang et al. (2023) (46)	China	VTE	LR(1)	Previous history of VTE/coronary heart disease/stroke, ECOG score of≥2, WBC> 11×10^9^/L, and ALB < 35g/L	0.754(0.698-0.811)	NR	Hosmer-Lemeshow test and calibration curve	Bootstrap	Nomogram and formula
Acute leukemia	Owattanapanich et al. (2023) (47)	Thailand	VTE or ATE	LR(1)	D-dimer>7000 µg FEU/L, platelet>40× 10^9^/L, and white blood cell level>15× 10^9^/L	0.83(0.75-0.90)	NR	Not report	Bootstrap	Risk score
Acute leukemia	Zhang et al. (2025) (50)	China	PICC-related thrombosis	LR (Model 1 of 2)	Puncture site, puncture times, catheter indwelling time, catheter- related complications, use of anticoagulant drugs and D-dimer level	0.775(0.690-0.846)	NR	Not report	None	Formula
Acute leukemia	Zhang et al. (2025) (50)	China	PICC-related thrombosis	RF (Model 2 of 2)	Puncture site, puncture times, catheter indwelling time, catheter- related complications, use of anticoagulant drugs and D-dimer level	0.860(0.785-0.916)	NR	Not report	None	random forest model
Mixed leukemia (ALL/AML/CML)	Fu et al. (2025) (51)	China	PICC-related thrombosis	LR(1)	Leukemia, number of catheters, history of catheterization, total parenteral nutrition, post-catheterization D-dimer, and post-catheterization fibrinogen	0.844(0.787-0.900)	0.794(0.698-0.890)	Hosmer-Lemeshow test and calibration curve	Bootstrap	Nomogram

LR, logistic regression; CR, cox regression; RF, random forest; NR, not report.

### 3.7 Risk of bias and applicability

**Figure 3:**
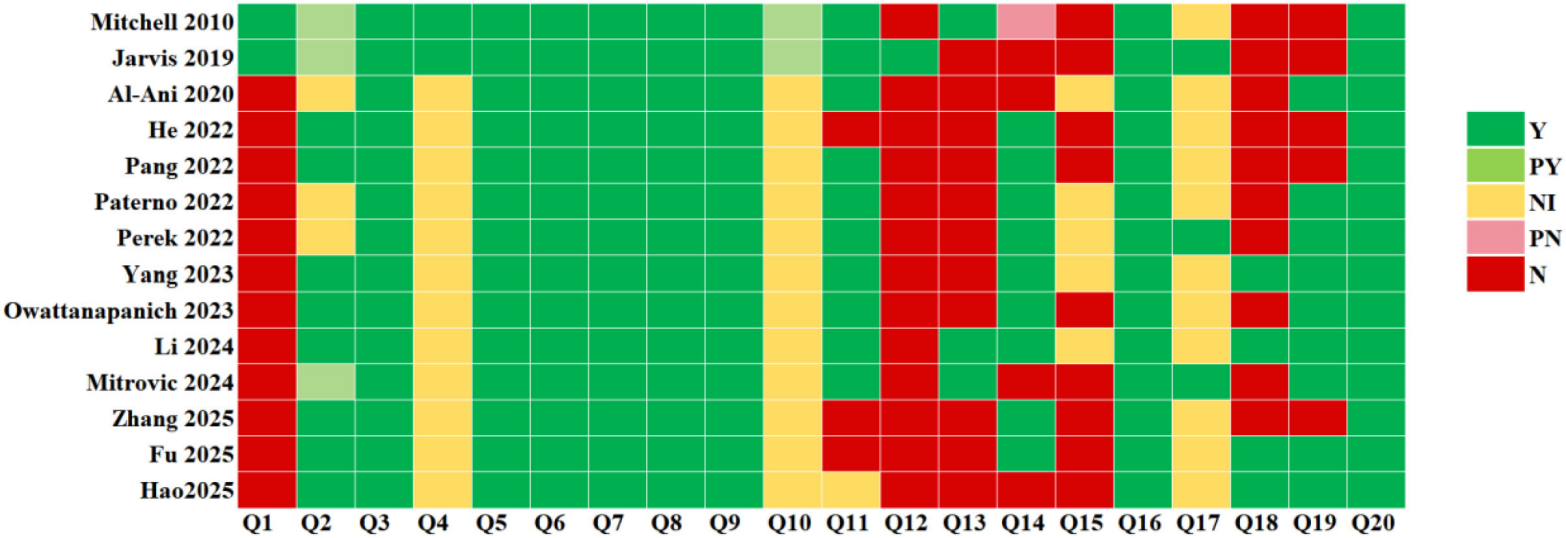
Evaluation of risk of bias using PROBAST. Y, yes; PY, probably yes; NI, no information; PN, probably not; N, no.

**Figure 4:**
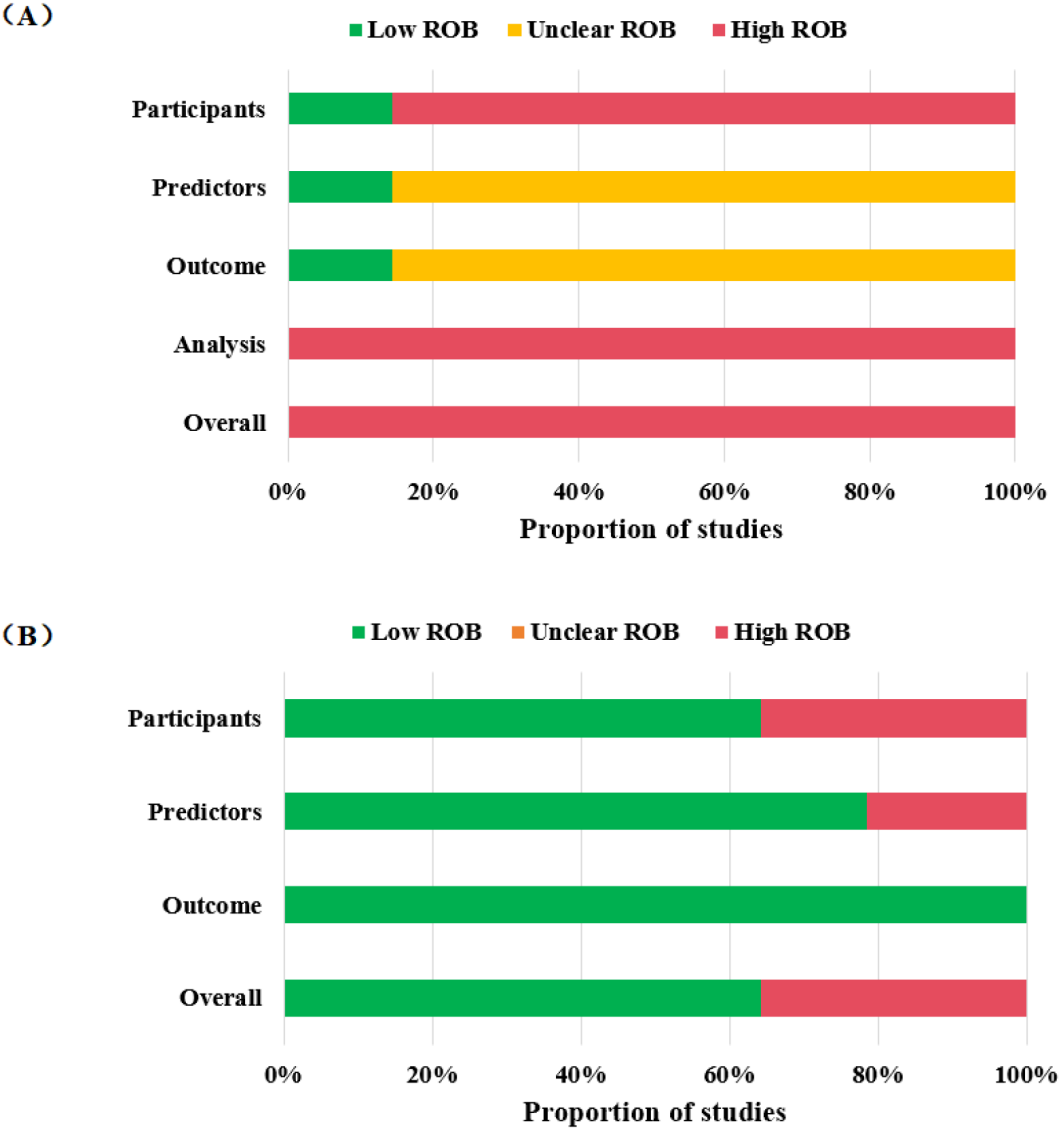
Rating of risk of bias and applicability. **(A)** Overall rate of risk of bias (ROB) based on PROBAST: low, green; high, red; unclear, yellow. **(B)** Overall rate of applicability based on PROBAST: low, green; high, red; unclear, yellow.

The PROBAST tool was used to evaluate the risk of bias and concerns regarding applicability across the included studies. The results are presented in **Table 5**, **Figures 3**, **4**.

**Table 5:** Results of bias and applicability risk assessment according to PROBAST.

Author(s) (year)	Risk of bias				Applicability			Overall	
	Participants	Predictors	Outcome	Analysis	Participants	Predictors	Outcome	Risk of bias	Applicability
Mitchell et al. (2010) (40)	L	L	L	H	H	L	L	H	H
Jarvis et al. (2019) (41)	L	L	L	H	L	L	L	H	L
Al-Ani et al. (2020) (42)	H	N	N	H	L	L	L	H	L
He et al. (2022) (43)	H	N	N	H	H	H	L	H	H
Pang et al. (2022) (44)	H	N	N	H	L	L	L	H	L
Paterno et al. (2022) (27)	H	N	N	H	L	L	L	H	L
Perek et al. (2022) (45)	H	N	N	H	H	L	L	H	H
Yang et al. (2023) (46)	H	N	N	H	L	L	L	H	L
Owattanapanich et al. (2023) (47)	H	N	N	H	L	L	L	H	L
Li et al. (2024) (48)	H	N	N	H	L	L	L	H	L
Mitrovic et al. (2024) (49)	H	N	N	H	L	L	L	H	L
Zhang et al. (2025) (50)	H	N	N	H	H	H	L	H	H
Fu et al. (2025) (51)	H	N	N	H	H	H	L	H	H
Hao et al. (2025) (52)	H	N	N	H	L	L	L	H	L

L, low risk of bias; H, high risk of bias; N, unclear risk of bias.

#### 3.7.1 Risk of bias

All 14 studies were assessed as having an overall high risk of bias. Within the ‘Participants’ domain, 12 studies—comprising 9 retrospective cohort studies (27, 42, 43, 45, 47, 49–52) and 3 case-control studies (44, 46, 48) were rated as high risk. In the ‘Predictor’ domain, 12 studies had an unclear risk of bias (27, 42–52) because they did not explicitly state whether predictors were assessed without knowledge of the outcome. Similarly, in the ‘Outcome’ domain, 12 studies had an unclear risk of bias (27, 42–52) due to insufficient information on whether outcomes were assessed without knowledge of the predictors. All studies were rated as high risk of bias in the ‘Analysis’ domain. Key reasons included: only one study met the recommended events per variable (EPV) threshold of at least 20 events per candidate predictor (41); 11 studies inappropriately handled continuous predictors by dichotomizing them (27, 41–47, 50–52); 9 studies handled missing data inadequately by excluding participants directly (40, 41, 43, 44, 47, 49–52), and 5 studies did not report the methods for handling missing data (27, 42, 45, 46, 48); only 4 studies fully reported both discrimination and calibration measures (46, 48, 51, 52);only 10 studies performed any form of internal and/or external validation (27, 40, 42, 45–49, 51, 52); and only 3 studies considered information related to data complexity (41, 45, 49).

#### 3.7.2 Applicability

Regarding the applicability issue, 5 studies raised high concerns. In the ‘Participants’ domain, 1 study was limited to leukemia patients receiving specific chemotherapy regimens (40), and 4 studies were limited to leukemia patients with PICC placement (43, 45, 50, 51); these 5 studies were thus rated as having high concerns regarding applicability for the review question. In the ‘Predictors’ domain, 3 studies were rated as having high concerns because predictors might have been measured after the thrombotic event had occurred (43, 50, 51). In the ‘Outcome’ domain, all studies were considered to have low concerns.

## 4 Discussion and analysis

This systematic review evaluated 14 studies comprising 16 risk prediction models for thrombosis in leukemia patients. By systematically examining model characteristics, performance reporting, risk of bias, and clinical relevance, we found that although several models reported moderate to good apparent discrimination and identified clinically plausible predictors, all included studies were judged to be at high risk of bias, and the overall evidence base is insufficient to support routine clinical implementation at this stage. The main methodological limitations were widespread, including small effective sample sizes with low EPV, suboptimal handling of continuous predictors, incomplete handling and reporting of missing data, and limited validation. Overall, these issues increase the possibility of overfitting and optimism, undermine calibration and transportability, and limit the real-world applicability of existing tools. Therefore, current models should be regarded as exploratory and should undergo rigorous prospective external validation before being used to guide thromboprophylaxis or other clinical decision-making. In the following sections, we discuss in detail the main sources of bias and limited applicability observed across studies.

The development of thrombosis risk prediction models in leukemia patients falls under prognostic modeling research. Prospective cohort studies are widely regarded as the optimal design for minimizing retrospective bias. However, among the 14 studies reviewed, only two adopted a prospective design, with the remaining 12 relying on retrospective cohort or case-control methodologies. A key distinction lies in the blinding process: the two prospective studies inherently ensured that predictors were assessed prior to outcome occurrence, whereas none of the retrospective studies explicitly described whether blinding was implemented—either in predictor assessment or in outcome evaluation. Proper blinding is essential to prevent information bias. If assessors are aware of thrombosis outcomes, they may overinterpret borderline predictor values, leading to inflated associations. Conversely, if clinicians know predictor values such as D-dimer levels, they might be more inclined to diagnose equivocal symptoms or imaging findings as thrombosis, increasing the likelihood of false-positive outcomes. Therefore, future efforts in model development should emphasize standardized implementation of blinding procedures.

Simultaneously, methodological deficiencies are also evident in sample size and variable handling. According to PROBAST guidance, prognostic model studies should ensure an “Events Per Variable (EPV) ≥ 20” to reduce overfitting (39). However, among the models included in this review, only one met this standard; the EPV in the remaining studies mostly ranged between 0.5 and 11.33, potentially leading to reduced generalizability of the models in external populations. Furthermore, 9 out of the 14 studies categorized continuous variables, which can lead to loss of information, reduced statistical power, and even mask non-linear relationships between predictors and the thrombotic outcome. To reduce categorization bias, we recommend that future studies use continuous variables in their original form during statistical analysis or consider non-linear regression techniques to fit the models.

Regarding missing data handling and model validation, among the 14 studies, 9 used complete-case analysis (direct deletion of cases with missing data), and 5 did not report the handling method. We recommend employing multiple imputation methods in future research to handle missing data, thereby enhancing the accuracy of reported results. Concerning model validation, only 9 studies performed internal validation, and only 1 conducted external validation, making it impossible to effectively assess the model’s overfitting risk and generalizability. Future studies should perform both internal and external validation, and consider temporal validation where necessary to evaluate the long-term stability of the models.

In the PROBAST assessment of concerns regarding applicability, 5 out of the 14 leukemia thrombosis risk prediction models raised high concerns. The core issues focused on insufficient participant representativeness and flaws in the temporal sequence of predictors. Regarding participants, 1 model was limited to patients on specific chemotherapy regimens, and 4 were confined to populations with PICC insertions. These limitations prevent the models from covering the heterogeneity of leukemia patients with different chemotherapy regimens and venous access choices in clinical practice, directly restricting their generalizability to the broader population. Regarding predictors, 3 studies potentially collected predictors after the thrombotic event had occurred, violating the core logic that predictors must precede the outcome, thereby rendering the models clinically non-predictive. Future research needs to include heterogeneous leukemia populations and strictly ensure the “predictor-outcome” temporal sequence to enhance the clinical promotion value of the models.

Another key consideration is generalizability. Nearly half of the included studies were conducted in China, and several reports were published in Chinese, including two dissertations. Differences in patient genetics, contemporary treatment protocols, supportive care standards, and thrombosis screening practices may limit the transportability of these models to other settings. To address concerns regarding evidence-source quality and indexing, we conducted restricted analyses excluding articles not published in English and excluding non–peer-reviewed dissertations. These analyses yielded consistent overarching conclusions but further highlighted the paucity of robust external validation across diverse populations.

From the analysis of predictors, the high-frequency factors identified across the 14 studies can be categorized into three core dimensions: patient characteristics (e.g., prior thrombosis history, comorbidities, age), treatment intervention factors (central venous catheter placement, chemotherapy/radiotherapy, use of hemostatic drugs), and disease-related indicators (e.g., D-dimer levels, platelet count, white blood cell count, ECOG score, leukemia type).

A prior history of thrombosis is a significant warning sign for recurrent thrombotic diseases. Leukemia patients with a history of thrombosis have approximately a 6-fold higher risk of subsequent venous or arterial thrombosis compared to those without such a history (33, 53). Comorbidities such as diabetes and hypertension indirectly increase thrombosis risk by damaging vascular endothelial function, promoting a hypercoagulable state, and activating platelets (54–56). Studies show that leukemia patients with ≥2 underlying chronic diseases have a significantly higher incidence of thrombotic events, by 1 to 3 times, compared to those with a single disease (57). Advancing age is closely related to alterations in coagulation mechanisms. Individuals over 65 years old exhibit a significantly increased thrombosis risk due to decreased vascular elasticity, increased activity of certain coagulation factors, and a chronic inflammatory state (58–60).

Central venous catheter placement is widely used in leukemia patients to administer chemotherapy drugs, provide parenteral nutrition, transfuse blood products, and facilitate blood sampling, while minimizing the discomfort of frequent venipuncture. Central venous catheters (CVCs) can be classified into four types: non-tunneled catheters, tunneled central catheters, fully implantable catheters, and peripherally inserted central catheters (PICCs) (61). CVCs can promote thrombosis due to mechanical injury to the vascular endothelium and adsorption of coagulation factors on the catheter surface, with PICC-related thrombosis incidence potentially being higher than other catheter types (62–64). Chemotherapy exacerbates abnormalities in the coagulation system of leukemia patients, increasing thrombotic risk. For instance, L-asparaginase, a common drug for ALL treatment, causes deficiencies in antithrombin, fibrinogen, protein C, and protein S, disrupting the physiological balance between anticoagulation and hemostatic pathways (65–68). Radiotherapy is important in specific scenarios for leukemia treatment, such as central nervous system leukemia, pre-transplant conditioning, or for tumor masses formed by leukemic cells. In a sub-analysis of the COMPASS-CAT study, radiotherapy was associated with an increased risk of venous thromboembolism (HR 2.47, 95% CI: 1.47-4.12), more pronounced in women than men (10.8% vs. 2.7%, p=0.03) (69). Some leukemia patients experience bleeding during chemotherapy. While hemostatic drugs can effectively control bleeding, their overuse can lead to a hypercoagulable state, increasing thrombosis risk (43).

D-dimer is a degradation product of fibrin broken down by plasmin. Abnormally elevated levels in the blood are a key marker reflecting secondary hyperfibrinolysis and a hypercoagulable state. Studies indicate that ALL patients with high D-dimer levels (≥4 μg/mL) have a 100-day cumulative incidence of venous or arterial thrombosis of 52.9%, compared to 13.8% in patients with low-to-moderate D-dimer levels (<4 μg/mL) (70). A platelet count exceeding 350×10^9^/L during hospitalization is associated with a 2.5-fold increased risk of VTE (71). In the outpatient setting, patients with a pre-chemotherapy platelet count ≥350×10^9^/L had a higher VTE incidence compared to those with a count <350×10^9^/L (3.98% vs. 1.37%) (72). The tumor microenvironment can induce thrombocytosis and platelet activation. Platelet activation leads to the release of prothrombotic molecules from alpha-granules and dense granules into the blood microenvironment and exposes activated sites for thrombin, correlating with increased thrombosis risk (73). When the peripheral blood WBC count in leukemia patients exceeds 50×10^9^/L, the risk of thrombosis increases, potentially related to blood stasis, stimulation of vascular endothelium by toxic metabolic oxidative products from leukemic cells, expression of procoagulant substances by leukemic cells, and release of inflammatory factors (48). The ECOG score is a tool to assess the functional status and ability to perform daily activities in cancer patients; typically, a score ≤2 is required for considering active treatment like chemotherapy. An ECOG score ≥2 indicates the patient is incapable of work; if combined with prolonged bed rest due to reduced mobility, slow blood flow, and poor functional status, it increases thrombosis risk (46). The risk of thrombosis in leukemia patients is closely related to the specific subtype, with APL having the highest risk, where abnormal promyelocytes simultaneously activate both the coagulation and fibrinolytic systems, predisposing to life-threatening disseminated intravascular coagulation (DIC) (74).

These predictive factors provide a clear basis for candidate variable selection in future model development. In clinical practice, dynamic monitoring based on these quantifiable indicators could potentially help healthcare staff identify high-risk individuals early and reduce thrombosis risk through preemptive interventions.

Notably, within our included evidence base, chronic leukemia was represented only by chronic myeloid leukemia (CML) in a single mixed-subtype cohort. Thrombosis in leukemia patients follows the classic Virchow triad—hemodynamic abnormalities, endothelial injury, and a hypercoagulable state, but the dominant contributing factors and management differ between acute and chronic forms. In acute leukemia, thrombosis is initiated primarily by the rapid release of tissue factor, microvesicles, and other procoagulant substances from leukemic cells, leading to the coagulation cascade and are often accompanied by disseminated intravascular coagulation (DIC) (75, 76). This creates a paradoxical risk of both bleeding and thrombosis, and management focuses on treating the underlying leukemia and controlling DIC with careful consideration of anticoagulation given high bleeding risk. In contrast, chronic leukemia develops over a prolonged course, where alterations in blood flow, persistent inflammation, and excessive or dysfunctional blood cells collectively induce a hypercoagulable state, predominantly resulting in macrovascular thrombosis (77, 78). Treatment therefore emphasizes thromboprophylaxis or therapeutic anticoagulation in conjunction with disease-directed therapy.

## 5 Limitations

This review has limitations. Although we implemented a comprehensive search across English and Chinese databases, relevant studies in other languages or unpublished literature may have been missed, which could contribute to reporting and publication bias. In addition, substantial clinical and methodological heterogeneity (e.g., leukemia subtype, outcome definition, predictors, and validation) precluded meaningful quantitative pooling. We therefore focused on a structured descriptive and comparative synthesis complemented by sensitivity analyses as robustness checks.

## 6 Future directions

Future research should prioritize prospective, multicenter cohorts with clearly defined time horizons and standardized outcome adjudication, ensuring that predictors are measured before outcome occurrence and that thrombosis screening practices are transparently reported. Rigorous model development should be supported by adequate sample size planning (including EPV considerations), principled handling of missing data (e.g., multiple imputation), and appropriate modeling of continuous predictors (avoiding unnecessary dichotomization and considering non-linear effects when justified).

External validation across diverse populations and healthcare systems should be considered a minimum requirement before any clinical implementation. When validation reveals calibration drift or reduced discrimination, model updating and recalibration strategies should be undertaken, and comparative studies that evaluate multiple candidate models within the same contemporary cohort are needed to identify the best-performing approach for specific clinical contexts.

Given subtype-specific differences in thrombosis mechanisms and treatment pathways, future models should either be developed and validated within specific leukemia subtypes or incorporate subtype in a way that is clinically interpretable and supported by sufficient data. In addition, models targeting catheter-related thrombosis should explicitly account for catheter type, insertion technique, catheter-related complications, and prophylaxis practices.

## 7 Conclusion

Current thrombosis risk prediction models for leukemia patients appear promising but remain insufficiently validated for routine clinical use. They should be considered exploratory and not used as stand-alone tools to guide thromboprophylaxis. Prospective multicenter external validation, transparent reporting, and model updating are essential to develop robust and transportable prediction models.
